# Comprehensive Evaluation of Quality and Antioxidant Capacity of Highbush Blueberries (*Vaccinium corymbosum*)

**DOI:** 10.3390/foods14183251

**Published:** 2025-09-19

**Authors:** Xiaoli Liu, Jia Zhang, Yindi Di, Haoliang Wan, Kunyu Wang, Jiyun Nie

**Affiliations:** 1 Laboratory of Quality & Safety Risk Assessment for Fruit (Qingdao), Ministry of Agriculture and Rural Affairs, Qingdao Key Laboratory of Modern Agriculture Quality and Safety Engineering, College of Horticulture, Qingdao Agricultural University, Qingdao 266109, China; 201901196@qau.edu.cn (X.L.); 18806370950@163.com (Y.D.); hl-w@outlook.com (H.W.); w18561747527@163.com (K.W.); 2 Xuzhou Institute of Agricultural Sciences of Xuhuai District of Jiangsu Province, Xuzhou 221131, China; zhangjia208@jaas.ac.cn; 3 Tongshan Test Station, Xuzhou Institute of Agricultural Sciences of the Xuhuai District of Jiangsu Province, Xuzhou 221121, China

**Keywords:** highbush blueberry, anthocyanin, antioxidant capacity, comprehensive evaluation

## Abstract

Blueberries, widely recognized for their antioxidant capacity, have driven rapid growth in China’s blueberry industry owing to their significant health benefits and economic value. However, a comprehensive evaluation for blueberry quality traits and antioxidant capacity remains lacking in China’s domestic research. This study systematically evaluated 26 highbush blueberry cultivars with consistent tree age and cultivation practices, which can better reflect cultivar-dependent trait variation. Key findings revealed Earliblue exhibited the highest soluble solid content (SSC) and the lowest titratable acidity (TA), while Bluechip had the most abundant vitamin C (VC). Glucose and fructose were the main components of soluble sugars in highbush blueberries, accounting for over 97% of the total sugars. Citric acid was the dominant organic acid in nearly all cultivars. Malvidin 3-*O*-galactoside, delphinidin 3-*O*-galactoside, delphinidin 3-*O*-arabinoside, malvidin 3-*O*-arabinoside and petunidin 3-*O*-galactoside were the most abundant anthocyanins. The 26 blueberry cultivars were graded into high-, medium- and low-anthocyanin content groups. Correlation analysis divided the 14 anthocyanins into two types: antioxidant-related anthocyanins and other anthocyanins. The five cultivars with the highest comprehensive evaluation scores were Sunrise, Bluegold, Elliott, Amblue and Briteblue. These results may establish empirical selection criteria for the selection and efficient utilization of high-quality blueberry cultivars.

## 1. Introduction

Blueberry, a member of the *Vaccinium* genus, has a strong antioxidant capacity, which contribute to its great medicinal and nutritional value [1]. The benefits include reducing eye strain and protecting vision, delaying brain cell aging and preventing neurodegenerative diseases, enhancing cardiorespiratory function and preventing cardiovascular diseases [2,3]. As a result, blueberry consumption has risen significantly in recent years [4,5]. In China, the cultivation area and total annual output of blueberries reached 66,400 hm^2^ and 347,200 t by the end of 2020, respectively [6]. Highbush blueberry (*Vaccinium corymbosum*) characterized by large fruit size, high yield and stronger antioxidant capacity, has become the predominant commercial varieties cultivated in northern China. The antioxidant activity of blueberries is mainly attributed to anthocyanins [7,8]. It is considered as an intrinsic antioxidant defense component in the modern food industry [9]. At present, the primary anthocyanidins that have been found in blueberries include delphinidin, malvidin, petunidin, cyanidin and peonidin, which are linked to various sugar moieties through the glycosidic bonds, resulting in the formation of anthocyanins such as malvidin 3-*O*-galactoside, delphinidin 3-*O*-arabinosid, and petunidin 3-*O*-galactoside [10,11]. Antioxidant capacity is an important quality characteristic of blueberries as well as a crucial index for selecting blueberry cultivars. In blueberries, the antioxidant activity is largely determined by the composition and content of anthocyanins [12,13]. The type and content of anthocyanins in blueberries are determined by genotype [14], and are also greatly influenced by the environment. Some cultivation conditions, such as orchard soil, plant age, treatment and harvest time can affect it in varying degrees [15].

In the comprehensive evaluation of blueberries, the highest soluble solid content (SSC) and the lowest titratable acidity (TA) are the basic evaluation indicators for variety selection and industrial application. Sugar and acid are the two basic components that affect the flavor and taste of blueberries. Soluble sugars, particularly glucose, sucrose and fructose, contribute to its sweet taste, while organic acids, especially citrate and malate, are the key factors to its sourness. Moderate acidity can enhance palatability, while high acidity levels often reduce fruit quality [16]. The vitamin C (VC) works synergistically with anthocyanins to enhance antioxidant activity. These parameters work together to directly control the palatability, storage tolerance and nutritional function of blueberries. Current research focuses on the effects of maturity on TA and SSC of blueberries [17], the dynamic changes of sugar and acid accumulation during ripening [18], and the levels of sugar and acid in blueberries from different regions [19]. It is important to pay attention to individual differences in sugar and acid composition and levels of blueberries grown in the same location.

In recent years, great research efforts have been made not only to improve fruit quality but also to enhance the nutritional value. Many published reports are usually focused on some famous blueberry cultivars (such as Duke, O’Neal, Bluecrop, Northland and Northblue), and the measured indices are largely inadequate [14,20]. Although there are a certain number of reports on phytochemicals and biological activities of blueberries, there is limited comparative studies on this topic among different varieties under the same climatic environment and cultivation practices [21,22,23,24]. This study included 26 highbush blueberry cultivars widely cultivated in northern China. All samples were collected from the same orchard to ensure consistency in tree age, climate and cultivation practices, which can better reflect genotype-dependent trait variation. We described the cultivar variability of highbush blueberries in terms of quality traits (SSC, TA, pH and VC), taste traits (major soluble sugars and organic acids), bioactive compounds (anthocyanins) and antioxidant activity (ABTS and FRAP). Analysis of variance (ANOVA), cluster analysis, correlation analysis and factor analysis were used to (1) compare the quality characteristics and differences of highbush blueberry cultivars; (2) focus on the composition characteristics of anthocyanins, and classify 26 blueberry cultivars based on this; and (3) explore the relationship between different traits, and comprehensively evaluate of quality of blueberries. This work may provide valuable implications for the selection and efficient utilization of high-quality blueberry cultivars.

## 2. Materials and Methods

**Figure 1:**
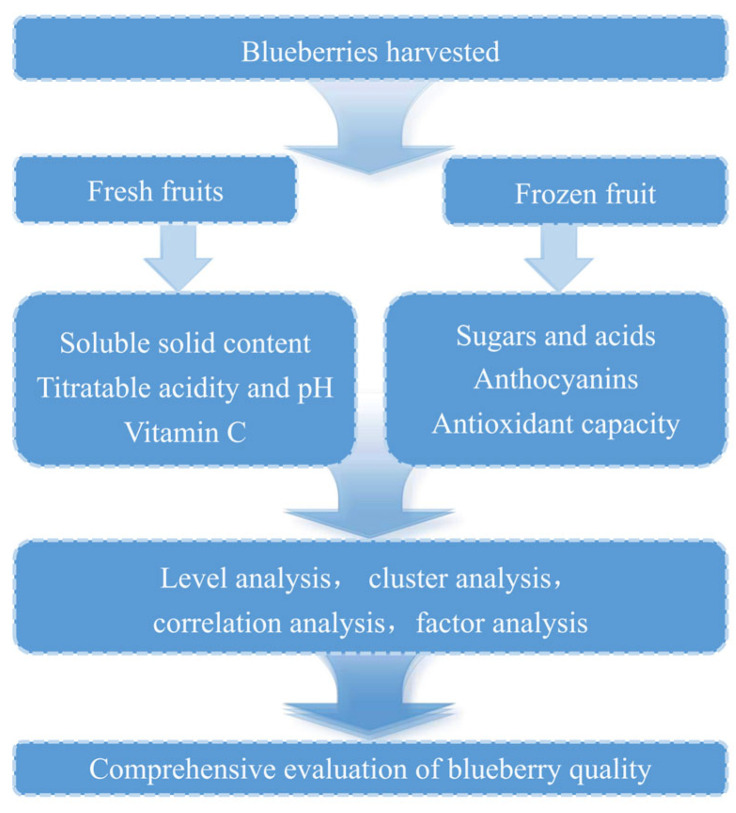
Experimental sequence.

The experimental procedure was carried out sequentially as illustrated in Figure 1.

### 2.1. Preparation of Fruit Materials

A total of 26 cultivars of highbush blueberry (*Vaccinium corymbosum*) with consistent tree age and management were included in this study, including Duke, Bluehaven, Hardyblue, Puru, Reka, Amblue, Brigitta, Olimpia, Earliblue, Bluecrop, Bluejay, Jubilee, Bluechip, Bonifacy, Sunrise, Bluegold, Patriot, Sweetheart, Coville, Spartan, Bluetta, Jersey, Lateblue, Elliott, O’Neal and Misty. All samples were collected from Weihai, Shandong Province of China (121°53′ E, 36°92′ N) in 2018. Lateblue and Elliott were harvested on 15 July, and the remaining of the cultivars were harvested in late June. The local climatic conditions during the growing season, including rainfall, temperature and sunshine duration, are summarized in Table 1. At the commercial maturity stage (fruits exhibited a full blue or dark blue coloration with a hard yet elastic texture), about 1 kg fruit of each cultivar were randomly selected from 15 trees. About 500 g fresh fruit were used for determination of fruit SSC, TA and VC. The remaining fruit were cut into small pieces, quickly frozen in liquid nitrogen, then ground into fine powder with a cryogenic grinder, stored at −20 °C. Soluble sugars, organic acids, anthocyanins and antioxidant activity were determined from July to August 2018, that is, within two months after harvest.

### 2.2. Determination of Fruit Quality Characteristics

SSC (°Brix) was determined by a digital refractometer (PR-101α, Atago, Tokyo, Japan) with the results presented as a percentage. TA analysis was based on the neutralization of acid with 0.1 mol/L NaOH, which was measured by an automatic potentiometric titrator (808 Titrando, Metrohm, Herisau, Japan). The results of this analysis were expressed as a percentage (%) of citric acid content, following the methodology outlined by Zhang et al. [19]. VC was titrated with 2,6-dichloroindophenol sodium salt hydrate (808 Titrando, Metrohm, Japan), and expressed as *L*-ascorbic acid mg/100 g of fresh fruit (FW).

### 2.3. Determination of Major Soluble Sugars and Organic Acids

Extractions of soluble sugars and organic acids were extracted using the methods reported by Zhang et al. [19] and Fan et al. [25] with some modifications. About 5 g of sample was weighed and mixed with 20 mL deionized water, and extracted by ultrasound at room temperature (25 °C) for 30 min. Then, the sample was centrifuged at 9000 r/min for 10 min. The ultrasonic extraction process was repeated twice. Then the extraction solution was transferred into a 50-mL volumetric flask, and made up to 50 mL with deionized water. Took 10 mL of the extraction solution and passed it through a Dionex Onguard™ II RP column (2.5 cc, Dionex, Sunnyvale, CA, USA) and then dilute the solution to 25 mL using deionized water. The extraction solution was diluted 50-fold for the determination of soluble sugars. The organic acids were determined without dilution of the extraction solution. A 0.22 μm cellulose membrane filter was used for filtration before analysis.

The determination of organic acids was conducted using high performance liquid chromatography (HPLC) (LC-10A, Shimadzu, Kyoto, Japan) with SPD-10A UV-VIS detector, and a C18 column (Ultimate LP-C_18_, 4.6 mm × 300 mm, 5 μm, Ultimate, Shanghai, China) following the method described by Martínez-Valdivieso et al. [26]. The eluate was analyzed via UV absorption at a wavelength of 210 nm. The operating conditions were as follows: the mobile phase was a 0.01 mol/L KH_2_PO_4_ solution; the flow rate was set at 0.5 mL/min; the column temperature was maintained at 40 °C; and the injection volume was 10 μL.

The determination of soluble sugars was conducted using an Ion Chromatograph (ICS-5000, Dionex, USA) equipped with a conductivity detector, a guard column (IonPac AG23, 4 mm × 50 mm, Dionex, USA) and an anion exchange analytical column (Dionex CarboPacTM PA10, 4 mm × 250 mm, Thermo Fisher Scientific, Waltham, MA, USA). The experimental procedure followed the methodology outlined by Zheng et al. [27]. The operational parameters were set as follows: the mobile phase consisted of a 0.2 mol/L NaOH solution; the flow rate was adjusted to 1.0 mL/min; the column temperature was maintained at 30 °C; and the injection volume was 10 μL. Data processing and instrument controlling were managed using the Chromeleon (6.80 SR9) software. Individual sugars and organic acids were identified and quantified based on the retention time and the peak area of corresponding standards, with the results stated as mg/g FW.

### 2.4. Extraction and HPLC-PDA Analysis of Anthocyanins

Extraction and High-Performance Liquid Chromatography-Photodiode Array Detector (HPLC-PDA) analysis of anthocyanins were performed according to the method reported by Li et al. [28] with minor modifications. 5 g of blueberry powder was weighed and extracted with 20 mL of pre-cooled 80% methanol solution at 4 °C for 12 h under stationary conditions. The extraction was carried out via ultrasound at room temperature (25 °C) for 15 min, followed by centrifugation at 9000 r/min for 10 min (Centrifuge CF16RX II, Hitachi, Tokyo, Japan). The ultrasound extraction was repeated twice. Then the extraction solution was transferred into a 50-mL volumetric flask, and brought up to volume with the same solvent. The sample was then filtered through a 0.22 μm nylon filter and used for HPLC-PDA (Shimadzu, Japan) and antioxidant analysis. Separation was achieved on an XSelect HSS T3 column (4.6 mm × 250 mm, 5 μm, Waters, Milford, MA, USA). The mobile phase consisted of 5% formic acid solution (A) and acetonitrile (B) as reported by Liu et al. [29]. The gradient program was as follows: 0–30 min, 6–8% B; 30–31 min, 8–9% B; 31–60 min, 9–12% B; 60–70 min, 12–100% B; 70–78 min, 6% B. The flow rate was set at 1 mL/min. The column temperature was maintained at 40 °C. The injection volume was 3 μL. Anthocyanins were identified and quantified based on the relative retention time and the peak area of standard, and the outcomes were presented as mg/100 g FW.

### 2.5. Antioxidant Activity Measurements

The antioxidant activity was determined using blueberry extract in part 2.4. Ferric-reducing antioxidant power (FRAP) assay was performed according to the literature Zhao et al. [30] using the Total Antioxidant Capacity Assay Kit (Beyotime Biotechnology, Shanghai,China). The FRAP reagent was prepared on the day of use and warmed to 37 °C prior to application. Blueberry extract (0.1 μL) was added to the mixture of 180 μL of the FRAP reagent and kept for 5 min. The absorbance of the mixture was measured at 593 nm using ELX-800UV Microplate Reader (BioTek, Winooski, VT, USA). The standard curve was constructed using a FeSO_4_ solution, and outcomes were reported as μM FeSO_4_/g of fruit.

Following the literature guidance of Zhao et al. [30], we used a total antioxidant capacity assay kit with the rapid ABTS method (Beyotime Biotechnology, China) to determine the Trolox equivalent antioxidant capacity (TEAC). Blueberry extract (0.1 μL) was added to the mixture of 170 μL ABTS working solution and 20 μL peroxidase working solution and maintained for 6 min. The absorbance of the mixture was measured at 405 nm using ELX-800UV Microplate Reader (BioTek, USA). Trolox was used as a standard, and the results were expressed as μM Trolox/g of fruit.

### 2.6. Statistical Analysis

All statistical analyses were carried out using SPSS 19.0 (IBM Corp., Armonk, NY, USA). Data were expressed as the mean ± standard deviation. Correlation between experimental variables was calculated using Pearson correlation. Significant differences were estimated using one-way ANOVA at the 5% significance level. K-means cluster analysis was used for anthocyanin classification of 26 blueberry cultivars. The comprehensive evaluation of blueberries quality was carried out by factor analysis, in which the extraction method was principal component analysis, and the rotation method was the varimax-rotation method. GraphPad Prism 8 (GraphPad Software, Inc., San Diego, CA, USA) was used for box plots and bar charts. Heat maps were created by using Heatmap tools in chiplot (https://www.chiplot.online/).

## 3. Results and Discussion

### 3.1. Quality Characteristics of Fruit

The fruit TA, SSC, VC content and pH are presented in Table 2. SSC is major attribute determining the acceptance of blueberries by consumers. In this study, the fruit SSC ranged from 10.8% to 20.0% with an average value of 13.6%, and exhibited significant (*p* < 0.05) differences among different cultivars. Notably, Amblue and Earliblue had the highest SSC (20.0% and 19.5%, respectively), suggesting that these two cultivars are more likely to have a savory taste. TA is also an important index affecting the fruit taste. In this study, TA ranged from 0.26% to 1.54%, exhibiting great variability among different cultivars as well, and Duncan’s multiple range test allocated all the blueberry cultivars into 15 groups in terms of TA. The lowest level of TA was found in O’Neal (0.26%) and Earliblue (0.28%), and the highest level was detected in Lateblue (1.57%) and Elliott (1.58%). The pH values of the tested blueberry cultivars ranged from 3.07 to 4.02, indicating that blueberries are generally acidic fruits. Among them, cultivars Puru, Reka, Bluegold and Lateblue exhibited the lowest pH values, while Amblue had the highest pH. Both titratable acidity (TA) and pH are indicators of acidity. Correlation analysis revealed a significant negative correlation between pH and TA (*r* = −0.568, *p* < 0.01). Regression analysis further showed a significant logarithmic relationship between pH and TA content (*p* < 0.05), with the fitted equation being pH = −0.284 ln (TA) + 3.225, and the coefficient of determination (*R^2^*) was 0.37. This result may be attributed to the differences in their measurement principles: TA reflects the total amount of acidic substances that can be neutralized by alkali, whereas pH characterizes the concentration of free hydrogen ions. Additionally, the partial dissociation of weak acids (such as citric acid) present in blueberries may also affect their correlation. The VC content among the 26 blueberry cultivars showed a significant variation, ranging from 8.1 to 15.6 mg/100 g FW. The cultivars were categorized into a high VC group (≥13.5 mg/100 g FW) including Bluechip, Amblue, Hardyblue and Patriot; a low VC group (<10.0 mg/100 g FW) comprising Bluecrop, Jubilee, Misty, O’Neal and Sweetheart; and a medium VC group (10.0–11.9 mg/100 g FW) containing all other cultivars. This analysis establishes a clear nutritional stratification for varietal selection, recommending prioritization of top-tier groups for high-VC application scenarios.

### 3.2. Major Soluble Sugars and Organic Acids

**Figure 2:**
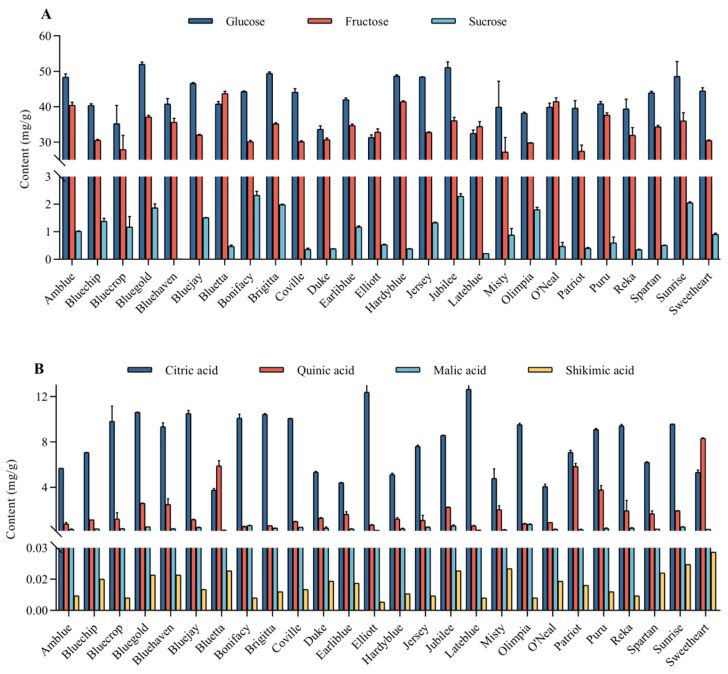
Levels of major soluble sugars (**A**) and organic acids (**B**) of 26 highbush blueberry cultivars (mg/g FW). Data were recorded as the mean ± SD (*n* = 3).

Soluble sugars and organic acids are important factors for the development of blueberry taste. The linear relationship, precision and repeatability of soluble sugars and organic acids were shown in Table S1. Figure 2 and Table S2 present the levels of major soluble sugars and organic acids in the fruit of the studied cultivars. All samples exhibited high levels of glucose and fructose, which ranged from 31.32 to 52.01 mg/g FW and from 27.21 to 43.72 mg/g FW, respectively. A particularly high glucose content was found in Bluegold (52.01 mg/g FW), while fructose content was especially high in Bluetta (43.72 mg/g FW). Compared with that of glucose and fructose, the content of sucrose was generally much lower, and a relatively higher sucrose content was found in Bonifacy (2.32 mg/g FW) and Jubilee (2.29 mg/g FW). Some studies identified fructose, glucose and sucrose in southern highbush blueberry, lowbush and several wild blueberry species, the first two sugars were the major soluble sugars in blueberry with similar content and relatively low sucrose concentration, which is consistent with the results of this study [31]. The levels of individual sugars (glucose, fructose, and sucrose) in blueberries change during ripening and storage, depending on sugar type, blueberry cultivars, and storage conditions. It was found that no sucrose was detected in Duke during harvesting and storage process, whereas in Elizabeth the sucrose content decreased during the ripening process and disappeared after storage at room temperature [32]. This phenomenon of sugar accumulation may be related to sucrose metabolism. Sucrose can be transported to fruit cells via symplast or apoplast pathways and is hydrolyzed to glucose and fructose by cell wall INV. During the ripening process of blueberry fruit, the expression of INV is up regulated, resulting in a significant increase in the content of glucose and fructose [31,33].

Citric acid, quinic acid, malic acid and shikimic acid were found in all analyzed blueberry samples, and the levels of organic acids were significantly (*p* < 0.05) different among blueberry cultivars (Figure 2 and Table S2). Citric acid was the dominant organic acid in most highbush blueberries, which ranged from 3.75 to 12.65 mg/g FW and was particularly high in Lateblue. Quinic acid ranged from 0.54 to 8.32 mg/g FW, and was the major organic acid in Sweetheart and Bluetta. The shikimic acid content of highbush blueberries was relatively low, which was no more than 0.03 mg/g FW (Sweetheart). Some studies have reported that the level of organic acids in the blueberries, found that citric acid accounts for 77–87% of total acid, quinic acid accounted for 4–11% of total acid, and only small amounts of shikimic acid have been detected in blueberries fruits [32,34,35]. This study is consistent with these results, citric acid (76.6% of total acids) was the dominant organic acid in most of the tested highbush blueberry cultivars. However, Sweetheart and Bluetta exhibited a quinic acid advantage due to their high levels of quinic acid (59.6% of total organic acids). Some fruits may have more than one dominant type of organic acids [36,37]. This is similar to a previous study about apricot, which revealed that apricot has both malic acid-dominant and critic acid-dominant accumulation patterns of organic acids [25]. Shikimic acid is one of the organic acid components in blueberry fruits. Although its content is lower than those of other organic acids, this finding is consistent with previous studies [34,38]. Wang et al. [34] reported that wild blueberries exhibit significantly higher levels of shikimic acid (4–10 times) compared to cultivated varieties, indicating that shikimic acid is an intrinsic metabolic component of the species. This suggests that shikimic acid may serve as a valuable marker for studying interspecific or varietal differences. In addition, we also detected low levels of malic acid in 26 blueberries, with a content of 0.21–0.73 mg/g FW, higher than shikimic acid, which has received less attention in other studies.

### 3.3. Characteristics of Anthocyanins

**Figure 3:**
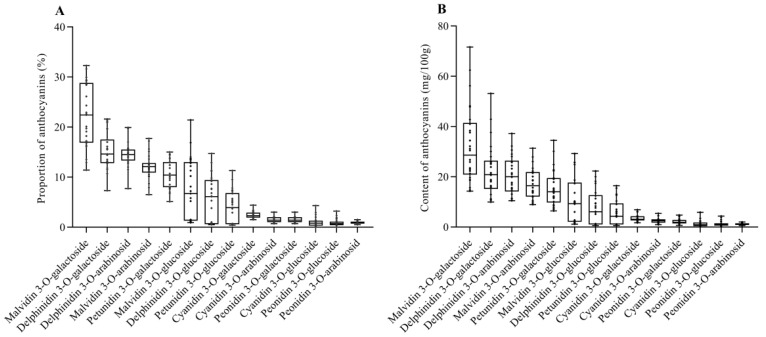
Anthocyanins proportions (**A**) and content (**B**) of highbush blueberry cultivars. The horizontal line inside the box is the median, and the lines outside are the maximum and minimum values, respectively. The solid circle represented the average. The box indicates the distribution of 50% of the data. Data were recorded as the mean ± SD (*n* = 26).

In order to further investigate the anthocyanin components in blueberry fruit, qualitative and quantitative analysis of anthocyanins in 26 blueberry cultivars were performed by HPLC-PDA. The linear relationship, precision and repeatability of anthocyanins were shown in Table S3. As a result, a total of 14 main anthocyanins were identified. The proportions and levels of anthocyanins in highbush blueberries are presented in Figure 3. The most abundant anthocyanins included malvidin 3-*O*-galactoside (33.12–71.61 mg/100 g FW, accounting for 22.3 ± 6.4%), delphinidin 3-*O*-galactoside (23.06–53.19 mg/100 g FW, accounting for 15.2 ± 3.8%), delphinidin 3-*O*-arabinoside (21.12–37.25 mg/100 g FW, accounting for 14.4% ± 2.4%), malvidin 3-*O*-arabinoside (17.55–31.36 mg/100 g FW, accounting for 12.1 ± 2.5%), and petunidin 3-*O*-galactoside (15.76–34.51 mg/100 g FW, accounting for 10.6 ± 2.9%), which is consistent with the findings of Skrede et al. [39]. Zhou et al. also determined anthocyanins in blueberries [40]. They found that malvidin-3-*O*-glucoside is the major anthocyanin, followed by malvidin-3-*O*-galactoside and petunidin-3-*O*-glucoside. Another study indicated that malvidin-3-*O*-glucoside, petunidin-3-*O*-glucoside and delphinidin-3-*O*-glucoside are dominant anthocyanin components in blueberries [41]. The result of Li et al. [42] indicated that malvidin-3-*O*-galactoside is the predominant anthocyanin, followed by malvidin-3-*O*-glucoside and delphinidin-3-*O*-galactoside in blueberries. These different findings can be attributed to the cultivar-dependent anthocyanin profiles and contents [41]. The composition and relative content of anthocyanins in different cultivars of blueberries are mainly determined by genetic factors [43,44]. Prior et al. [45] detected 16 anthocyanins in lowbush blueberries (*Vaccinium angustifolium*), and the dominant anthocyanin components include malvain-3-*O*-glucoside, malvain-3-*O*-galactoside, petunidin-3-*O*-glucoside and delphinidin-3-*O*-galactoside. Guo et al. [46] studied the wild blueberry in Northeast China and found that wild blueberry contains 14–16 kinds of anthocyanins, mainly including delphinidin, petunidin and malvain derivatives.

According to Yan et al. [47], Pearson correlation analysis performed across 20 blueberry genotypes over two harvest seasons (2019 and 2020) revealed that although some correlations reached statistical significance—such as between lightness and peonidin glycoside concentration, chroma and anthocyanin arabinoside concentration, and hue angle and total anthocyanin concentration—all correlation coefficients were small (*r* < 0.4). These results suggest that the inherent differences in anthocyanin concentration or composition among mature blueberry samples are not strongly reflected in static color parameters. It is important to emphasize that blueberry surface color is influenced by a combination of factors other than anthocyanins. For instance, Holcroft and Kader [48] demonstrated that vacuolar pH can significantly modulate anthocyanin coloration, while Lara et al. [49] highlighted the role of cuticular waxes in affecting fruit appearance. These non-anthocyanin factors may obscure the contribution of anthocyanin differences to perceived color variation. Future studies would benefit from integrating transcriptomic, metabolomic, and colorimetric approaches to quantitatively assess the relative contributions of anthocyanin concentration and structure, vacuolar pH, cuticular wax components (such as β-diketones), and other potential factors to blueberry color. Such multidisciplinary efforts could further elucidate the interactive networks governing fruit pigmentation and appearance.

The total anthocyanin content was calculated as the accumulation of 14 individual anthocyanins, and was found to vary significantly (*p* < 0.05) among cultivars, which is consistent with published research results [14]. The average content ranged from 83.3 mg/100 g FW (Misty) to 252.02 mg/100 g FW (Sunrise). Twelve cultivars exceeded the average level of total anthocyanin content (144.25 mg/100 g FW). Seven cultivars had a total anthocyanin content lower than 100 mg/100 g FW, and only two cultivars had a total anthocyanin content higher than 200 mg/100 g FW. Cultivation conditions are important factors affecting the accumulation of anthocyanins in blueberry fruits. Studies have shown that moderate water stress can significantly increase the content of delphinidin-3-acetylhexoside in the fruits [50]. Plastic cover cultivation reduces anthocyanin levels compared to open-field cultivation, with different plastic film colors altering anthocyanin composition [51]. Genotype is also a key factor determining the anthocyanin content in blueberries. A study conducted by Rossi et al. [52] on 71 blueberry cultivars revealed that the Duke and Earliblue varieties had the highest total anthocyanin content, while the Puru variety had the lowest, which highly consistent with the results of this study. Research by Chunhong Zhang et al. [53] also indicated that the Duke variety had the highest anthocyanin content. However, their study showed high anthocyanin accumulation in O’Neal, whereas our study detected only 73.88 ± 1.56 mg/100 g, below average. This difference may relate to environmental adaptability. O’Neal belongs to southern highbush blueberry and is more suitable for growing in the environment of southern China, while all the blueberry samples in this study were grown in northern China. Fruit developmental stage also markedly affects anthocyanin accumulation. A study by Sun et al. [54] found that there are obvious differences in the main components of anthocyanins at different fruit developmental stages: cyanidin is the main component at the green fruit stage, delphinidin is the main component at the red fruit stage, and malvidin is the main component at the blue fruit stage; meanwhile, the total anthocyanin content of fruits at the blue fruit stage is also significantly higher than those at the green, pink, red and purple fruit stages.

**Figure 4:**
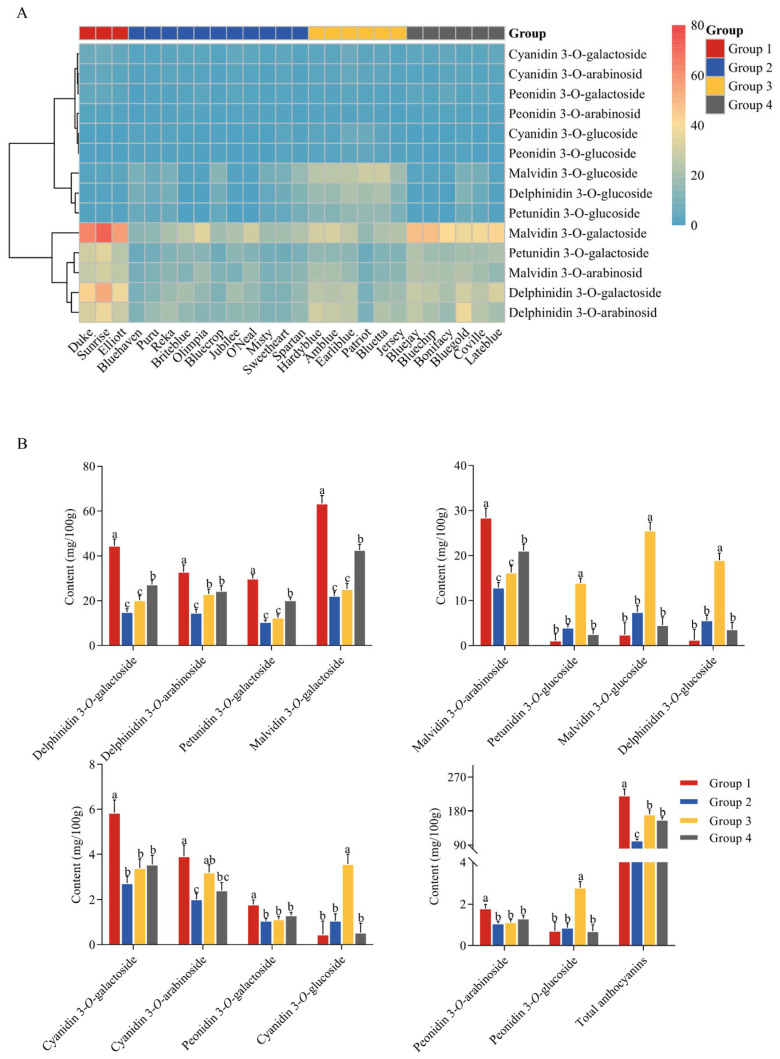
(**A**): Heat map of cluster diagram of the average content of individual anthocyanin in 26 blueberry cultivars. Data were recorded as the average content of individual anthocyanin (*n* = 3), the content of each anthocyanin (mg/100 g FW) was reflected as the intensity of color. (**B**): ANOVA results of anthocyanins contents among different groups. Data were recorded as the mean ± SD (*n* = 3). ANOVA and the Duncan test were performed. When *p* < 0.05, the values with significant differences are indicated by different letters.

Cluster analysis has become one of the commonly used methods in plant breeding, genetic relationship and origin studies, resource utilization and classification. In this study, 26 blueberry cultivars were divided into 4 groups according to anthocyanin type and content. ANOVA was used to compare the concentrations of anthocyanins among different groups. The results are shown in Figure 4. Group 1 included Duke, Sunrise and Elliott, in which delphinidin 3-*O*-galactoside, malvidin 3-*O*-galactoside, malvidin 3-*O*-arabinoside, cyanidin 3-*O*-galactoside and peonidin 3-*O*-arabinoside contents were higher than the other groups. Group 2 consisted of Bluehaven, Puru, Reka, Briteblue, Olimpia, Bluecrop, Jubilee, O’Neal, Misty, Sweetheart and Spartan, except petunidin 3-*O*-glucoside, malvidin 3-*O*-glucoside, delphinidin 3-*O*-glucoside, cyanidin 3-*O*-glucoside and peonidin 3-*O*-glucoside, the contents of other anthocyanins were lower than those of the other three groups. Group 3 included Hardyblue, Amblue, Earliblue, Patriot, Bluetta and Jersey, which had higher contents of petunidin 3-*O*-glucoside, malvidin 3-*O*-glucoside, delphinidin 3-*O*-glucoside, cyanidin 3-*O*-glucoside and peonidin 3-*O*-glucoside than the other groups. Group 4 covered cultivars Bluejay, Bluechip, Bonifacy, Bluegold, Coville and Lateblue. The contents of delphinidin 3-*O*-galactoside, petunidin 3-*O*-galactoside, malvidin 3-*O*-galactoside and malvidin 3-*O*-arabinoside were higher than those of groups 2 and 3. The order of total anthocyanins contents was ranked as Group 1 (220.72 ± 30.38 mg/100 g FW) > group 3 (171.49 ± 27.14 mg/100 g FW) > group 4 (157.26 ± 17.42 mg/100 g FW) > group 2 (101.45 ± 14.06 mg/100 g FW), that is, group 1 was high anthocyanin level, group 3 and group 4 were medium anthocyanin levels, and group 2 had low level of anthocyanin.

The health benefits of anthocyanins are closely linked to their structural types and concentrations. Malvidin has been shown to induce apoptosis in hepatic stellate cells, indicating that blueberries rich in malvidin could act as a functional food source for supporting liver health [55]. Furthermore, malvidin significantly enhances calcium deposition in mesenchymal stem cells, thereby facilitating osteogenic and chondrogenic differentiation [56]. It also mitigates sepsis-associated encephalopathy in mice by restoring mitochondrial function in the brain [57]. Malvidin-3′-*O*-glucoside exhibits antioxidant activity in neuronal cells by inhibiting acetylcholinesterase and alleviating oxidative stress [58]. Additionally, both malvidin-3-*O*-glucoside and malvidin-3-*O*-galactoside have been shown to markedly reduce free fatty acid-induced lipid accumulation [59]. Based on our findings, we recommend that future research focus on Group 1 cultivars—including Duke, Sunrise, and Elliott, which are rich in malvidin-3-*O*-galactoside—and Group 3 cultivars, such as Hardyblue, Amblue, Earliblue, Patriot and Bluetta (rich in malvidin-3-*O*-glucoside), for the development of functional or nutritional foods enriched with malvidin. Cyanidins also exhibit potent antioxidant properties [60]. Their anti-obesity effects are mediated by activating the phospholipase C–inositol-1,4,5-trisphosphate (IP3) pathway and intracellular Ca^2+^ signaling [61]. Moreover, cyanidin-3-*O*-glucoside and its phenolic metabolites help ameliorate intestinal diseases by modulating the mucosal immune system [62]. Zhu et al. [63] reported that purified delphinidin-3-*O*-β-glucoside and cyanidin-3-*O*-β-glucoside from blueberries significantly reduce inflammatory markers, and also improve lipid profiles by lowering LDL-cholesterol and elevating HDL-cholesterol. These findings suggest that Group 3 cultivars may hold significant potential for use in functional foods targeting cardiovascular and metabolic health. Anthocyanins exhibit structure-specific biological mechanisms and show promising applications in the prevention and adjuvant treatment of chronic metabolic disorders, inflammatory diseases, and neurological conditions. Further research on their mechanisms of action and clinical translation will facilitate the development of anthocyanin-based functional foods and pharmaceuticals.

### 3.4. Antioxidant Capacity

**Figure 5:**
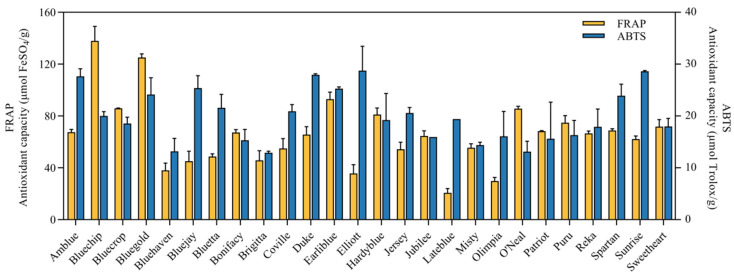
FRAP and ABTS of 26 highbush blueberry cultivars. Data were recorded as the mean ± SD (*n* = 3). FRAP values were expressed as μmol FeSO_4_/g. ABTS values were expressed as μmol Trolox/g.

The antioxidant activity of blueberries can be attributed to the bioactive compounds with antioxidant properties. In this study, the antioxidant capacity of highbush blueberry extracts was determined by ABTS and FRAP assays. As shown in Figure 5 and Table S5, the ABTS value ranged from 12.9 to 28.7 μmol Trolox/g. Relatively higher ABTS values were found in Elliott (28.7 μmol Trolox/g), Sunrise (28.6 μmol Trolox/g) and Duke (28.0 μmol Trolox/g), while low ABTS values were found in Bluehaven (13.2 μmol Trolox/g), O’Neal (13.1 μmol Trolox/g) and Brigitta (12.9 μmol Trolox/g). The differences in ABTS value were statistically significant (*p* < 0.05) among various highbush blueberry cultivars. The three cultivars with the highest ABTS values (Elliott, Sunrise, Duke) all belong to Group 1, which was previously identified to be rich in malvidin-3-*O*-galactoside and malvidin 3-*O*-arabinosid. This correlation suggests that the strong free radical scavenging capacity (reflected by ABTS assay) of Group 1 cultivars may be driven by malvidin—consistent with previous findings that malvidin derivatives inhibit oxidative stress [58]. The FRAP value ranged from 20.8 (Lateblue) to 137.9 μmol FeSO_4_/g (Bluechip), exhibiting great variability across different cultivars, and Duncan’s multiple range test allocated all the blueberry cultivars into 16 groups. The inconsistent results between ABTS and FRAP assays (such as Elliott ranks top in ABTS but not in FRAP, while Bluechip ranks top in FRAP but not in ABTS) reflect the diversity of antioxidant mechanisms among cultivars [64,65]. Group 1 cultivars (Elliott, Sunrise) excel in radical scavenging (ABTS), making them suitable for applications targeting “free radical-induced cell damage”; Group 4 cultivar Bluechip excels in reducing power (FRAP), which is more relevant for “metal ion-related oxidative stress”. We also observed that Bluechip, Earliblue and Bluegold, which possess both high ABTS and FRAP, may have better antioxidant value for consumers. This distinction helps readers understand the targeted antioxidant value of each cultivar, rather than simply judging “good or bad” by a single index.

### 3.5. Correlation Analysis of Blueberries Indicators and Antioxidant Activity in Blueberries

**Figure 6:**
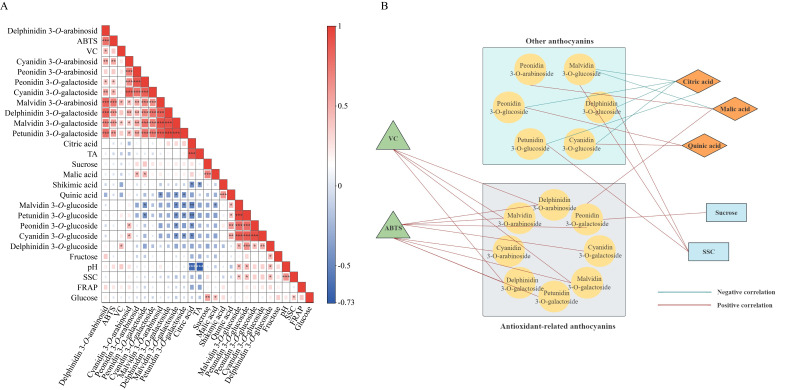
(**A**): Pearson correlation heat-map of the 26 blueberry indicators. Blue and red represent negative and positive correlations, respectively. The gradient color bar denoted the level of correlation from low to high. *, **, *** represent the significance of Pearson’s correlation, *p* < 0.05, *p* < 0.01, *p* < 0.001. (**B**): Relationship between anthocyanins contents and some indexes of highbush blueberries.

Pearson correlation analysis was performed on 26 blueberry indicators, and the results were shown in Figure 6. This approach facilitates the identification of the critical bioactive components. It is important to note that antioxidant activity is generally not attributable to a single compound, but rather arises from the synergistic or antagonistic interactions among multiple constituents. Correlation analysis helps elucidate these complex interactive effects. ABTS exhibited significant (*p* < 0.001) positive relationships with some anthocyanin components, including delphinidin 3-*O*-arabinoside (*r* = 0.813), delphinidin 3-*O*-galactoside (*r* = 0.742), malvidin 3-*O*-arabinoside (*r* = 0.719), petunidin 3-*O*-galactoside (*r* = 0.671), malvidin 3-*O*-galactoside (*r* = 0.657), cyanidin 3-*O*-galactoside (*r* = 0.521) and cyanidin 3-*O*-arabinoside (*r* = 0.500). VC showed significant (*p* < 0.05) positive correlations with malvidin 3-*O*-arabinoside, delphinidin 3-*O*-arabinoside, malvidin 3-*O*-galactoside and delphinidin 3-*O*-glucoside. Citric acid showed significant (*p* < 0.05) negative correlations with malvidin 3-*O*-glucoside, petunidin 3-*O*-glucoside, peonidin 3-*O*-glucoside and cyanidin 3-*O*-glucoside. Interestingly, these four anthocyanins all contain glucosides, possibly because citric acid is an influential factor in anthocyanin synthesis with glucosides. In addition, quinic acid showed significant (*p* < 0.05) negative correlations with malvidin 3-*O*-arabinoside, malvidin 3-*O*-galactoside and petunidin 3-*O*-galactoside. Although the analysis revealed no direct correlation between shikimic acid and antioxidant activity, shikimic acid serves as a key intermediate in plant secondary metabolic pathways. It acts as a precursor to numerous bioactive compounds, and through downstream oxidative modifications, gives rise to secondary metabolites such as flavonoids [66,67]. Thus, shikimic acid is suggested to contribute to antioxidant activity indirectly via its precursor role, rather than through direct involvement.

In the breeding process, the selection of target characteristics needs to take into account the possible negative correlation between traits, so as to avoid losing those fruit characteristics that are negatively correlated with the selected characteristics [68]. No correlation was observed between FRAP and anthocyanin content in this study. This result is consistent with that of a previous study [69], possibly because ABTS is more strongly affected by anthocyanins, while FRAP is more significantly affected by proanthocyanidins. Moreover, there was a positive correlation between VC content and ABTS (*p* < 0.05) (Figure 6A). According to the results of correlation analysis, the 14 anthocyanins were divided into two types: antioxidant-related anthocyanins and other anthocyanins (Figure 6B). In the development of blueberry cultivars with high antioxidant activity, priority should be given to the cultivars with high content of antioxidant-related anthocyanins.

### 3.6. Comprehensive Quality Evaluation of Blueberries Using Factor Analysis and Cultivar Ranking

The use of multivariate statistical analysis methods for comprehensive evaluation of multiple indicators is widely used in fruit research [70,71]. Factor analysis was performed for VC, SSC, TA, major soluble sugars, organic acids, FRAP, ABTS and anthocyanins of 26 blueberry cultivars. The data were standardized before the analysis. In this study, the cumulative variance contribution rate of the first five factors reached 80.9%, which could represent most of the information of the test indicators (Table 3). The first three factors (F1, F2 and F3) mainly explained the variance in 14 anthocyanins and ABTS, which explained 32.6%, 22.6% and 9.8% of the variance, respectively. F4 had major contributions from malic acid, glucose and sucrose. And TA and citric acid mainly contributed to F5. these two factors were concerned with sweet and sour tastes of blueberries, and accounted for 9% and 7% of the variance, respectively.

With the ratio of variance contribution and cumulative variance contribution of each factor as the weight, the comprehensive score model of main inner quality of blueberry was as follows: Q = 0.403 F1 + 0.279 F2 + 0.121 F3 + 0.111 F4 + 0.087 F5. Table 4 showed the score of blueberries on five factors. The higher the score on this factor, the higher the level of the representative indicator. Sunrise, Bluegold and Amblue had the highest score in F1, F2 and F3, respectively, indicating that these cultivars had highest anthocyanins contents and ABTS radical scavenging activity. Bluegold has high malic acid, glucose and sucrose contents (representative factor of F4), while Briteblue has the high TA and citric acid contents (representative factor of F5). The top five blueberry cultivars with comprehensive score were Sunrise, Bluegold, Elliott, Amblue and Briteblue, indicating that these cultivars have advantages in comprehensive quality.

### 3.7. Discussion on the Intake of Blueberries

Based on findings from multiple studies, the amount of blueberries required to produce significant health benefits varies depending on the specific health outcome and the form in which they are consumed. For improvements in postprandial glycaemic response, Bell et al. [72] reported that a dose of 310 mg of anthocyanins (equivalent to 235–555 g of fresh blueberries), significantly extended blood glucose availability in a dose-dependent manner, which is beneficial for metabolic health. Regarding plasma antioxidant capacity, Mazza et al. [73] demonstrated that consuming 100 g of lyophilized (freeze-dried) blueberry powder, containing 1.2 g of anthocyanins, led to a notable improvement in antioxidant status. For cardiometabolic benefits in individuals with type 2 diabetes, Stote et al. [74] found that daily intake of 22 g of freeze-dried blueberry powder over an 8-week period positively affected parameters such as insulin sensitivity and lipid profiles. We support that the effective dosage depends on the form of blueberry (fresh vs. freeze-dried), the duration of consumption, and the specific health objective. It should be noted that the exact fresh weight equivalent may vary based on blueberry variety and growing conditions. Freeze-dried blueberries offer a practical alternative for achieving high anthocyanin intake without the need for large amount, making them suitable for daily supplementation.

## 4. Conclusions

This study evaluated the quality traits (SSC, TA, VC and pH), taste traits (major soluble sugars and organic acids), bioactive compounds (anthocyanins), and antioxidant activity (ABTS and FRAP) of 26 highbush blueberry cultivars collected from the same orchard with consistent tree age and cultivation practices, which can better reflect genotype-dependent trait variation. The results clearly demonstrated that cultivar has significant influence on the chemical components of highbush blueberries. Glucose and fructose are the major soluble sugars, while citric acid and quinic acid are dominant organic acids in highbush blueberries. The proportions of some anthocyanins are stable in the studied highbush blueberry cultivars, and the most abundant anthocyanins include malvidin 3-*O*-galactoside, delphinidin 3-*O*-galactoside, delphinidin 3-*O*-arabinoside, malvidin 3-*O*-arabinoside and petunidin 3-*O*-galactoside. The 26 blueberry cultivars were graded into high-, medium-, and low-anthocyanin content groups by cluster analysis. In addition, the antioxidant capacity detected by ABTS is significantly positively correlated with the anthocyanin content, and 14 anthocyanins were divided into two types: antioxidant-related anthocyanins and other anthocyanins. Factor analysis evaluated that the top five cultivars with comprehensive scores were Sunrise, Bluegold, Elliott, Amblue and Briteblue.

## Figures and Tables

**Table 1:** Summary of monthly and annual averages of climate data.

Parameter	January	February	March	April	May	June	Annual
Average rainfall (mm)	3.1	5.1	32.2	54.2	75.9	99.7	791.5
Average temperature (℃)	−3	−1.4	5.8	11.5	17.2	21.8	12.2
Sunshine duration (h)	131.7	164.1	199.1	213.8	232.6	176.2	2234.6

**Table 2:** Titratable acidity (TA), soluble solids content (SSC), vitamin C (VC) content and pH in 26 blueberry cultivars.

Cultivars	TA (%)	SSC (%)	VC (mg/100 g)	pH
Amblue	0.34 ± 0.01 ^m^	20.0 ± 0.4 ^a^	14.5 ± 0.2 ^b^	4.02 ± 0.05 ^n^
Bluechip	0.32 ± 0.02 ^m,n^	11.2 ± 0.1 ^l,m^	15.6 ± 0.5 ^a^	3.38 ± 0.01 ^g,h^
Bluecrop	0.55 ± 0.03 ^k^	14.2 ± 0.1 ^e,f^	9.7 ± 0.1 ^h,i^	3.19 ± 0.06 ^c,d^
Bluegold	0.66 ± 0.01 ^h^	11.8 ± 0.1 ^j,k,l^	10.8 ± 0.8 ^d,e,f,g^	3.09 ± 0.03 ^a^
Bluehaven	1.09 ± 0.01 ^d^	12.3 ± 0.1 ^i,j^	11.2 ± 0.1 ^d,e,f^	3.10 ± 0.03 ^a,b^
Bluejay	0.56 ± 0.03 ^j,k^	16.2 ± 0.1 ^c^	11.6 ± 0.1 ^c,d^	3.38 ± 0.00 ^g,h^
Bluetta	0.63 ± 0.02 ^h,i^	14.8 ± 0.1 ^d,e^	10.3 ± 0.1 ^d,e,f,g^	3.43 ± 0.05 ^h,i^
Bonifacy	0.55 ± 0.01 ^k^	12.0 ± 0.2 ^j,k^	11.9 ± 1.6 ^c,d^	3.19 ± 0.01 ^c,d^
Brigitta	0.57 ± 0.01 ^i,j,k^	17.4 ± 0.1 ^b^	11.5 ± 0.2 ^c,d^	3.25 ± 0.01 ^e^
Coville	1.18 ± 0.04 ^b^	12.8 ± 0.0 ^h,i^	11.0 ± 0.6 ^d,e,f,g^	3.23 ± 0.03 ^d,e^
Duke	0.62 ± 0.01 ^h,i,j^	12.1 ± 0.1 ^j,k^	12.3 ± 0.7 ^c^	3.46 ± 0.01 ^i,j^
Earliblue	0.28 ± 0.01 ^n,o^	19.5 ± 0.2 ^a^	12.4 ± 0.8 ^c^	3.79 ± 0.03 ^m^
Elliott	1.58 ± 0.02 ^a^	10.8 ± 0.3 ^m^	12.3 ± 0.3 ^c^	3.16 ± 0.04 ^c^
Hardyblue	0.74 ± 0.01 ^g^	13.7 ± 0.1 ^f,g^	14.5 ± 0.6 ^b^	3.56 ± 0.03 ^k,l^
Jersey	0.92 ± 0.01 ^e^	14.5 ± 0.1 ^e^	10.0 ± 0.3 ^g,h,i^	3.56 ± 0.03 ^k,l^
Jubilee	0.48 ± 0.05 ^l^	13.8 ± 0.1 ^f,g^	9.1 ± 0.1 ^i,j,k^	3.31 ± 0.01 ^f^
Lateblue	1.57 ± 0.02 ^a^	11.5 ± 0.2 ^k,l,m^	10.8 ± 0.1 ^d,e,f,g^	3.09 ± 0.03 ^a^
Misty	0.35 ± 0.01 ^m^	12.0 ± 0.2 ^j,k^	8.6 ± 0.2 ^j,k^	3.47 ± 0.05 ^i,j^
Olimpia	0.56 ± 0.01 ^j,k^	15.3 ± 0.2 ^d^	10.3 ± 0.4 ^d,e,f,g^	3.38 ± 0.03 ^g^
O’Neal	0.26 ± 0.02 ^o^	11.2 ± 0.1 ^l,m^	9.5 ± 0.5 ^h,i,j^	3.56 ± 0.02 ^l^
Patriot	0.53 ± 0.01 ^k,l^	12.3 ± 1.4 ^i,j^	13.5 ± 0.1 ^b^	3.41 ± 0.01 ^g,h^
Puru	1.16 ± 0.01 ^b,c^	12.9 ± 0.1 ^h,i^	10.2 ± 0.6 ^f,g,h,i^	3.07 ± 0.01 ^a^
Reka	1.11 ± 0.02 ^c,d^	11.1 ± 0.1 ^m^	11.5 ± 0.5 ^c,d^	3.07 ± 0.05 ^a^
Spartan	0.84 ± 0.10 ^f^	14.5 ± 0.2 ^e^	11.3 ± 0.4 ^c,d,e^	3.51 ± 0.01 ^j,k^
Sunrise	0.52 ± 0.02 ^k,l^	12.8 ± 0.1 ^h,i^	12.3 ± 0.1 ^c^	3.51 ± 0.00 ^j,k,l^
Sweetheart	0.47 ± 0.01 ^l^	13.3 ± 0.1 ^g,h^	8.1 ± 0.4 ^k^	3.14 ± 0.02 ^b,c^

Data were recorded as the mean ± SD (*n* = 3). ANOVA and the Duncan test were performed. When *p* < 0.05, the values with significant differences are indicated by different letters.

**Table 3:** Rotational component matrix of factor analysis.

Index	F1	F2	F3	F4	F5
VC (*x*1)	0.254	0.419	0.037	−0.024	−0.070
SSC (*x*2)	0.388	0.008	−0.019	0.295	−0.210
TA (*x*3)	−0.170	0.016	−0.031	−0.006	0.951
Quinic acid (*x*4)	0.420	−0.182	0.132	−0.049	0.027
Malic acid (*x*5)	−0.257	0.018	0.256	0.837	0.088
Shikimic acid (*x*5)	−0.031	−0.010	0.071	0.070	−0.315
Citric acid (*x*7)	−0.370	0.112	−0.017	0.117	0.868
Glucose (*x*8)	0.126	0.055	−0.172	0.801	−0.048
Fructose (*x*9)	0.161	0.128	−0.059	0.007	−0.161
Sucrose (*x*10)	−0.294	0.054	0.209	0.771	0.082
FRAP (*x*11)	0.164	0.135	−0.023	0.185	−0.219
ABTS (*x*12)	0.251	0.888	0.119	−0.040	−0.052
Delphinidin 3-O-galactoside (*x*13)	−0.229	0.830	0.393	−0.013	0.127
Delphinidin 3-O-glucoside (*x*14)	0.944	−0.037	−0.178	−0.047	−0.154
Cyanidin 3-O-galactoside (*x*15)	−0.073	0.466	0.849	−0.028	−0.009
Delphinidin 3-O-arabinosid (*x*16)	0.099	0.900	0.145	0.089	0.083
Cyanidin 3-O-glucoside (*x*17)	0.923	−0.184	0.174	−0.124	−0.075
Petunidin 3-O-galactoside (*x*18)	−0.349	0.800	0.407	0.031	0.122
Cyanidin 3-O-arabinosid (*x*19)	0.367	0.369	0.809	−0.021	−0.053
Petunidin 3-O-glucoside (*x*20)	0.949	−0.041	−0.168	−0.051	−0.176
Peonidin 3-O-galactoside (*x*21)	−0.281	0.433	0.801	0.212	−0.012
Peonidin 3-O-glucoside (*x*22)	0.946	−0.102	0.190	−0.088	−0.033
Malvidin 3-O-galactoside (*x*23)	−0.385	0.802	0.377	0.001	0.036
Peonidin 3-O-arabinosid (*x*24)	−0.077	0.283	0.868	0.172	−0.015
Malvidin 3-O-glucoside (*x*25)	0.931	−0.045	−0.206	−0.072	−0.207
Malvidin 3-O-arabinosid (*x*26)	−0.206	0.878	0.278	0.104	−0.068
Variance (%)	32.6	22.6	9.8	9	7
Cumulative variance (%)	32.6	55.1	64.9	73.9	80.9

**Table 4:** Scores of principal factors and synthetic ranks of different blueberry cultivars.

Cultivars	F1 Score	F2 Score	F3 Score	F4 Score	F5 Score	Comprehensive Score	Rank
Amblue	1.239	−1.151	2.255	−0.141	0.062	0.441	4
Bluechip	1.228	−1.355	−1.486	1.241	1.303	0.188	9
Bluecrop	−0.689	0.041	−0.690	−0.442	−0.177	−0.414	23
Bluegold	0.477	1.766	0.086	3.044	0.102	1.042	2
Bluehaven	−0.772	0.299	−0.152	−0.480	0.716	−0.237	18
Bluejay	0.555	0.708	1.020	−0.879	−1.080	0.353	7
Bluetta	−0.153	−0.371	0.859	−0.522	−1.470	−0.247	19
Bonifacy	0.134	0.253	−0.359	0.256	1.747	0.262	8
Briteblue	−0.392	0.392	1.943	−0.373	2.483	0.361	5
Coville	0.116	0.563	−0.340	−0.258	−0.919	0.054	11
Duke	1.381	−1.339	−1.561	−0.467	−0.946	−0.140	17
Earliblue	0.864	−1.488	1.122	0.000	−1.324	−0.046	13
Elliott	1.522	1.411	−1.298	−1.253	−0.269	0.687	3
Hardyblue	1.026	−0.995	0.453	1.341	0.248	0.361	6
Jersey	−0.476	−0.146	0.628	−0.595	−1.297	−0.335	21
Jubilee	−1.890	0.320	0.898	0.911	−0.759	−0.529	24
Lateblue	−0.371	1.189	−0.643	−1.522	0.251	−0.043	12
Misty	−1.385	−1.360	−1.067	−0.590	0.438	−1.094	26
Olimpia	−0.356	0.085	0.359	−1.638	1.440	−0.133	16
O’Neal	−1.356	−1.736	−0.679	0.913	−0.016	−1.013	25
Patriot	0.430	0.214	−0.904	−0.344	0.456	0.125	10
Puru	−0.968	0.824	−0.107	0.910	−0.345	−0.102	15
Reka	−0.196	0.643	−1.093	0.361	−0.662	−0.049	14
Spartan	−0.412	−0.809	0.334	−0.241	0.725	−0.315	20
Sunrise	1.783	1.322	0.566	0.224	0.117	1.191	1
Sweetheart	−1.338	0.722	−0.144	0.545	−0.824	−0.366	22

## Data Availability

The original contributions presented in this study are included in the article/Supplementary Materials. Further inquiries can be directed to the corresponding author.

## Supplementary Materials

The following supporting information can be downloaded at: https://www.mdpi.com/article/10.3390/foods14183251/s1, Table S1: Methodological validation of the established sugar and acid component detection method; Table S2: Content comparison of major soluble sugars and organic acids among 26 blueberry cultivars; Table S3: Methodological validation of the established anthocyanins detection method; Table S4: Level comparison of anthocyanins among 26 blueberry cultivars; Table S5: Level comparison of antioxidant capacity among 26 blueberry cultivars.

## Abbreviations

The following abbreviations are used in this manuscript:

FRAP	Ferric-Reducing Antioxidant Power
HPLC	High Performance Liquid Chromatography
HPLC-PDA	High-Performance Liquid Chromatography-Photodiode Array Detector
RP	Reversed-Phase
SSC	Soluble Solid Content
TA	Titratable Acidity
TEAC	Trolox Equivalent Antioxidant Capacity
VC	Vitamin C
