# Supplementary material for: Comprehensive Evaluation of Quality and Antioxidant Capacity of Highbush Blueberries (Vaccinium corymbosum)
Source: Foods. 2025 Sep 19;14(18):3251. doi: 10.3390/foods14183251 (PMC12469497; doi:10.3390/foods14183251)
Supplement: Supplementary file 1 [file foods-14-03251-s001.zip › foods-3846679-supplementary.pdf]

Table S1: Methodological validation of the established sugar and acid component detection method.

Components	Linear range ($mg\cdot L^{-1}$)	Regression equations	r^2	LOD ($mg\cdot L^{-1}$)	Precision RSD (%)	Repeatability RSD (%)
Glucose	1—50	$y = 1.9484x + 1.4131$	0.9986	0.03	0.23—2.52	0.98—5.61
Fructose	1—50	$y = 1.4117x + 1.4417$	0.9985	0.02	0.28—3.64	0.57—5.89
Sucrose	0.5—35	$y = 0.9498x + 0.7073$	0.9993	0.03	1.04—5.66	1.23—8.37
Quinic acid	1—100	$y = 618.96x - 4585.9$	0.9998	0.30	0.15—6.36	1.23—6.56
Malic acid	1—100	$y = 1004.7x - 2427$	0.9996	0.10	0.32—8.07	1.56—9.09
Shikimic acid	0.5—50	$y = 171008x - 9358$	0.9997	0.03	0.53—8.55	2.89—9.38
Citric acid	1—100	$y = 1313x - 3625.1$	0.9996	0.30	0.25—5.07	0.25—6.08

Table S2: Content comparison of major soluble sugars and organic acids among 26 blueberry cultivars.

Cultivars	Soluble sugars (mg/g)			Organic acids (mg/g)			
	Glucose	Fructose	Sucrose	Quinic acid	Malic acid	Shikimic acid	Citric acid
Amblue	48.37±0.88[abcd]	40.44±0.80[bc]	1.02±0.01[gh]	0.77±0.13[ijk]	0.30±0.03[hij]	0.01±0.01[kl]	5.69±0.01[hi]
Bluechip	40.38±0.45[efgh]	30.56±0.24[ijk]	1.38±0.11[ef]	1.14±0.01[hijk]	0.34±0.01[fghi]	0.02±0.01[fg]	7.05±0.02[g]
Bluecrop	35.22±5.17[ij]	27.94±3.95[klm]	1.17±0.38[fg]	1.20±0.57[hij]	0.35±0.01[efgh]	0.01±0.01[lm]	9.81±1.34[cde]
Bluegold	52.01±0.62[a]	37.09±0.48[de]	1.87±0.14[cd]	2.59±0.01[d]	0.52±0.01[c]	0.02±0.01[e]	10.61±0.02[b]
Bluehaven	40.80±1.52[efgh]	35.64±1.12[def]	0.39±0.01[ij]	2.49±0.50[de]	0.34±0.02[fghi]	0.02±0.01[ef]	9.34±0.34[def]
Bluejay	46.55±0.28[bcd]	32.03±0.19[hij]	1.51±0.01[e]	1.16±0.03[hijk]	0.47±0.01[cd]	0.01±0.01[i]	10.50±0.28[bc]
Bluetta	40.78±0.67[efgh]	43.72±0.65[a]	0.47±0.05[ij]	5.88±0.46[b]	0.21±0.01[k]	0.02±0.01[cd]	3.75±0.14[m]
Bonifacy	44.27±0.19[cdef]	30.10±0.37[ijklm]	2.32±0.15[a]	0.54±0.02[k]	0.64±0.01[b]	0.01±0.01[klm]	10.10±0.35[bcd]
Brigitta	49.42±0.39[ab]	35.16±0.33[defg]	1.98±0.02[cd]	0.62±0.01[jk]	0.40±0.01[ef]	0.01±0.01[ij]	10.43±0.06[bc]
Coville	44.10±1.02[def]	30.10±0.29[ijklm]	0.36±0.04[ij]	0.98±0.02[ijk]	0.49±0.01[c]	0.01±0.01[i]	10.07±0.01[bcd]
Duke	33.63±0.98[jk]	30.72±0.48[ijk]	0.37±0.01[ij]	1.28±0.04[hi]	0.41±0.09[de]	0.01±0.01[gh]	5.34±0.06[ij]
Earliblue	41.98±0.51[efgh]	34.67±0.47[defgh]	1.17±0.04[fg]	1.62±0.23[gh]	0.32±0.03[ghi]	0.01±0.01[h]	4.40±0.03[klm]
Elliott	31.32±0.75[jk]	32.86±0.88[fghi]	0.53±0.02[ij]	0.69±0.01[ijk]	0.21±0.01[k]	-	12.38±0.78[a]
Hardyblue	48.66±0.31[abc]	41.42±0.24[ab]	0.39±0.01[ij]	1.19±0.14[hij]	0.34±0.05[fghi]	0.01±0.01[jk]	5.12±0.12[ijk]
Jersey	48.42±0.04[abcd]	32.74±0.19[fghij]	1.33±0.02[ef]	1.08±0.45[hijk]	0.49±0.02[c]	0.01±0.01[kl]	7.60±0.10[g]
Jubilee	51.09±1.60[a]	36.06±0.93[de]	2.29±0.09[ab]	2.25±0.01[def]	0.60±0.08[b]	0.02±0.01[cd]	8.58±0.02[f]
Lateblue	29.91±0.95[k]	32.24±1.38[ghij]	0.33±0.01[j]	0.61±0.02[jk]	0.21±0.01[k]	0.01±0.01[klm]	12.65±0.36[a]
Misty	39.88±7.30[fgh]	27.21±4.12[m]	0.88±0.24[h]	2.03±0.34[defg]	0.25±0.03[jk]	0.02±0.01[c]	4.78±0.84[jkl]
Olimpia	38.15±0.33[hi]	29.80±0.06[jklm]	1.80±0.09[d]	0.78±0.04[ijk]	0.73±0.03[a]	0.01±0.01[klm]	9.51±0.12[de]
O'Neal	39.91±1.08[fgh]	41.46±1.07[ab]	0.47±0.14[ij]	0.90±0.01[ijk]	0.30±0.02[hij]	0.01±0.01[gh]	4.07±0.21[lm]
Patriot	39.63±2.13[ghi]	27.46±1.73[lm]	0.40±0.03[ij]	5.84±0.26[b]	0.28±0.02[ij]	0.01±0.01[h]	7.07±0.19[g]
Puru	40.87±0.63[efgh]	37.65±0.62[cd]	0.60±0.21[i]	3.76±0.39[c]	0.38±0.05[efg]	0.01±0.01[i]	9.09±0.09[ef]
Reka	39.37±2.77[hi]	31.97±2.17[hij]	0.34±0.02[j]	1.93±0.91[efg]	0.41±0.03[de]	0.01±0.01[kl]	9.42±0.11[de]
Spartan	43.96±0.43[defg]	34.35±0.40[efgh]	0.51±0.01[ij]	1.66±0.25[fgh]	0.32±0.01[ghi]	0.02±0.01[de]	6.20±0.05[h]
Sunrise	48.59±4.16[abc]	35.99±2.35[de]	2.05±0.04[bc]	3.86±0.02[c]	0.51±0.01[c]	0.02±0.01[b]	9.56±0.01[de]

Sweetheart	44.48±0.91[cde]	30.42±0.22[ijkl]	0.90±0.05[h]	8.32±0.03[a]	0.30±0.01[hij]	0.03±0.01[a]	5.30±0.21[ij]

Data were recorded as the mean ± SD (n=3). ANOVA and Duncan test was performed. When $p < 0.05$, the values of the same column which are significantly different are indicated by different letters.

Table S3: Methodological validation of the established anthocyanins detection method.

Anthocyanins	Linear range ($mg \cdot L^{-1}$)	Regression equations	r^2	LOD ($mg \cdot L^{-1}$)	Precision RSD (%)	Repeatability RSD (%)
Delphinidin 3-*O*-galactoside	0.5—100	$y = 15782x - 14148$	0.9999	0.17	0.1—1.23	1.32—4.78
Delphinidin 3-*O*-glucoside	0.5—50	$y = 11510x - 5391.5$	0.9999	0.10	0.13—1.52	1.68—5.41
Cyanidin 3-*O*-galactoside	0.5—100	$y = 21819x - 33104$	0.9995	0.10	0.8—3.33	1.99—8.20
Delphinidin 3-*O*-arabinosid	0.5—100	$y = 9553.5x - 7620.4$	0.9997	0.20	1.04—3.66	0.02—4.92
Cyanidin 3-*O*-glucoside	0.5—50	$y = 13753x - 7220.8$	0.9991	0.10	0.57—4.34	1.75—9.17
Petunidin 3-*O*-galactoside	0.5—80	$y = 12946x - 23077$	0.9991	0.17	0.98—2.61	1.40—4.56
Cyanidin 3-*O*-arabinosid	0.5—50	$y = 11311x - 3984.8$	0.9999	0.17	0.53—5.87	0.09—3.07
Petunidin 3-*O*-glucoside	0.5—50	$y = 12535x - 9094.5$	0.9996	0.10	0.23—6.57	0.85—4.01
Peonidin 3-*O*-galactoside	0.5—50	$y = 9931.1x - 2401$	0.9999	0.17	0.19—3.23	0.64—8.70
Peonidin 3-*O*-glucoside	0.5—50	$y = 13080x - 6615.9$	0.9998	0.17	0.02—2.25	2.93—8.47
Malvidin 3-*O*-galactoside	0.5—100	$y = 11361x - 11867$	0.9994	0.10	1.02—4.65	1.92—6.37
Peonidin 3-*O*-arabinosid	1.0—50	$y = 7581.3x - 8361.8$	0.9997	0.17	1.23—3.12	1.28—4.09
Malvidin 3-*O*-glucoside	0.5—100	$y = 11758x - 18922$	0.9997	0.17	1.55—5.23	0.40—5.78
Malvidin 3-*O*-arabinosid	0.5—100	$y = 9747.2x - 4769.5$	1.0000	0.10	0.56—4.21	1.88—4.40

Table S4: Level comparison of anthocyanins among 26 blueberry cultivars.

Cultivars	Delphinidin 3-*O*-galactoside	Delphinidin 3-*O*-glucoside	Cyanidin 3-*O*-galactoside	Delphinidin 3-*O*-arabinosid	Cyanidin 3-*O*-glucoside	Petunidin 3-*O*-galactoside	Cyanidin 3-*O*-arabinosid	Petunidin 3-*O*-glucoside	Peonidin 3-*O*-galactoside	Peonidin 3-*O*-glucoside	Malvidin 3-*O*-galactoside	Peonidin 3-*O*-arabinosid	Malvidin 3-*O*-glucoside	Malvidin 3-*O*-arabinosid	Total anthocyanins
Amblue	23.918±0.035 *h*	17.406±0.044 *d*	2.745±0.026 *j*	28.096±0.145 *d*	2.264±0.022 *d*	15.017±0.015 *gh*	2.524±0.011 *h*	12.686±0.017 *c*	1.540±0.069 *m*	2.066±0.050 *d*	32.169±0.015 *hi*	0.874±0.152 *l-k*	25.608±0.017 *bc*	21.737±0.154 *e*	188.651±0.736 *de*
Bluechip	25.868±0.512 *g*	0.925±0.023 *m-o*	2.757±0.045 *j*	21.532±0.037 *h*	- *m*	19.114±0.024 *ef*	1.571±0.065 *mn*	0.930±0.015 *jk*	1.940±0.006 *jk*	- *k*	- *d*	1.020±0.104 *i-k*	2.112±0.122 *k-m*	22.315±0.062 *de*	100.083±0.992 *i*
Bluecrop	11.244±0.181 *m*	8.339±0.143 *i*	2.111±0.206 *l*	13.076±0.594 *m*	1.211±0.089 *gh*	7.523±0.089 *m-o*	1.353±0.114 *o*	5.581±0.042 *h*	0.951±0.029 *n-p*	0.917±0.041 *f*	18.644±0.099 *n*	0.838±0.026 *lm*	13.603±0.237 *f*	12.089±0.209 *k*	97.478±2.088 *n*
Bluegold	30.084±0.296 *e*	11.366±0.149 *g*	3.102±0.011 *f-h*	37.212±0.610 *a*	1.347±0.042 *fg*	19.468±0.042 *e*	2.761±0.363 *fg*	7.021±0.076 *f*	1.675±0.396 *lm*	1.278±0.007 *e*	37.611±0.121 *g*	1.133±0.037 *g-j*	9.817±0.138 *g-i*	23.455±0.181 *d*	187.329±2.461 *e*
Bluehaven	9.980±0.1470 *m*	9.476±0.071 *h*	1.805±0.011 *mn*	11.237±0.082 *n*	1.718±0.010 *e*	6.412±0.010 *o*	1.411±0.058 *no*	6.076±0.006 *g*	0.726±0.061 *p*	1.192±0.109 *e*	14.333±0.026 *o*	0.758±0.072 *mn*	10.315±0.037 *g*	9.357±0.113 *lm*	84.797±0.814 *op*
Bluejay	28.028±0.111 *f*	1.056±0.016 *m-o*	3.556±0.001 *e*	25.902±0.245 *e*	0.345±0.058 *l*	22.737±0.152 *d*	2.412±0.123 *hi*	1.036±0.016 *jk*	3.299±0.029 *d*	0.867±0.111 *fg*	48.248±0.400 *d*	1.592±0.022 *cd*	2.057±0.096 *k-m*	23.450±0.104 *d*	164.584±1.269 *g*
Bluetta	18.358±0.367 *j*	22.234±0.392 *a*	3.239±0.058 *fg*	23.203±0.493 *g*	3.487±0.112 *b*	11.497±0.112 *j*	3.247±0.258 *c*	16.423±0.094 *a*	1.640±0.576 *lm*	2.943±0.151 *c*	22.273±0.089 *lm*	0.980±0.002 *lk*	29.168±0.828 *a*	13.634±0.375 *j*	172.325±3.903 *f*
Bonifacy	21.552±0.253 *i*	0.738±0.03 *no*	4.401±0.058 *d*	17.486±0.311 *j*	0.415±0.007 *kl*	17.880±0.007 *f*	3.041±0.045 *de*	0.792±0.053 *jk*	4.096±0.019 *c*	0.916±0.052 *f*	41.011±0.151 *ef*	1.833±0.027 *b*	1.676±0.019 *l-n*	21.476±0.172 *e*	137.312±1.204 *j*
Brigitta	20.715±0.608 *i*	0.718±0.068 *no*	2.800±0.066 *ij*	15.655±0.506 *kl*	0.313±0.001 *l*	14.479±0.001 *g-i*	1.725±0.476 *lm*	0.656±0.066 *k*	1.680±0.054 *lm*	0.156-±0.032 *k*	- *jk*	0.851±0.033 *lm*	1.373±0.101 *mn*	12.291±0.532 *k*	73.253±2.541 *mn*
Coville	25.338±0.412 *gh*	5.992±0.035 *k*	3.111±0.014 *f-h*	24.417±0.212 *f*	0.789±0.030 *i*	19.503±0.030 *e*	2.173±0.089 *jk*	4.568±0.008 *i*	2.477±0.281 *hi*	0.508±0.072 *ij*	38.388±0.011 *fg*	1.193±0.069 *gh*	9.141±0.005 *i*	20.122±0.232 *f*	157.720±1.514 *h*
Duke	42.905±1.461 *b*	1.327±0.031 *m*	6.306±0.126 *b*	32.208±0.853 *b*	0.429±0.033 *kl*	30.138±0.033 *b*	4.468±1.058 *b*	1.165±0.091 *j*	4.490±0.024 *b*	0.657±0.276 *g-i*	62.420±0.032 *b*	2.028±0.001 *a*	2.364±0.065 *kl*	27.880±0.838 *b*	218.780±4.921 *b*
Earliblue	25.330±0.038 *gh*	22.322±0.069 *a*	6.029±0.035 *c*	25.736±0.034 *e*	5.782±0.127 *a*	13.916±0.127 *hi*	5.463±0.056 *a*	15.504±0.019 *b*	2.675±0.189 *gh*	3.799±0.008 *b*	26.745±0.047 *j*	1.487±0.006 *de*	25.791±0.431 *b*	17.136±0.222 *h*	197.717±1.407 *c*
Elliott	37.662±0.626 *c*	1.209±0.032 *mn*	4.307±0.113 *d*	29.058±0.604 *d*	0.414±0.012 *kl*	24.903±0.012 *c*	2.738±0.662 *g*	1.094±0.058 *jk*	2.766±0.046 *fg*	0.786±0.047 *f-h*	56.239±0.317 *c*	1.363±0.025 *ef*	2.756±0.264 *k*	26.059±0.542 *c*	191.354±3.360 *de*
Hardyblue	25.130±0.268 *gh*	18.232±0.197 *c*	3.028±0.032 *gh*	30.634±0.219 *c*	2.534±0.001 *c*	15.435±0.001 *g*	2.933±0.073 *ef*	13.107±0.049 *c*	1.699±0.065 *k-m*	2.156±0.021 *d*	31.144±0.014 *i*	1.052±0.022 *h-j*	24.866±0.305 *c*	21.697±0.194 *e*	193.647±1.459 *cd*
Jersey	18.811±0.341 *j*	13.655±0.225 *e*	2.359±0.055 *k*	19.898±0.196 *i*	1.495±0.049 *f*	11.935±0.049 *j*	1.774±0.266 *l*	10.374±0.001 *d*	1.062±0.233 *no*	1.352±0.006 *e*	23.488±0.066 *kl*	0.748±0.026 *mn*	18.714±0.205 *d*	14.187±0.262 *j*	139.847±1.978 *j*
Jubilee	21.013±0.165 *i*	0.946±0.007 *m-o*	4.318±0.054 *d*	15.201±0.150 *l*	0.413±0.075 *kl*	14.110±0.075 *hi*	2.794±0.011 *fg*	0.734±0.048 *jk*	2.989±0.018 *ef*	0.540±0.043 *h-j*	22.407±0.234 *l*	1.431±0.048 *e*	1.177±0.002 *n*	9.965±0.328 *lm*	98.036±0.974 *n*
Lateblue	32.048±2.059 *d*	1.163±0.089 *m-o*	4.367±0.195 *d*	20.116±1.400 *i*	0.310±0.009 *l*	22.338±0.009 *d*	2.493±1.834 *h*	1.068±0.155 *jk*	2.405±0.112 *i*	0.587±0.138 *h-j*	42.754±0.045 *e*	1.071±0.152 *h-j*	2.260±0.133 *kl*	15.671±1.225 *i*	148.651±7.506 *i*
Misty	11.006±0.245 *m*	5.145±0.035 *l*	1.716±0.001 *n*	10.614±0.164 *n*	0.647±0.008 *ij*	8.449±0.008 *lm*	0.963±0.141 *p*	4.090±0.008 *i*	0.971±0.075 *n-p*	0.583±0.041 *h-j*	18.842±0.144 *n*	0.825±0.048 *m*	9.404±0.251 *hi*	10.042±0.149 *lm*	83.298±1.317 *p*
Olimpia	15.880±1.917 *k*	0.635±0.065 *o*	3.126±0.311 *f-h*	13.092±1.549 *m*	0.34±0.012 *l*	13.563±0.012 *i*	2.090±1.638 *k*	0.700±0.249 *jk*	3.195±0.035 *de*	0.384±0.427 *j*	34.426±0.086 *h*	1.584±0.147 *cd*	1.369±0.183 *mn*	18.720±2.361 *g*	109.105±8.995 *l*
O'Neal	16.345±0.237 *k*	0.628±0.032 *o*	3.534±0.023 *e*	14.55±0.0610 *l*	0.31±0.002 *l*	12.266±0.002 *j*	2.294±0.306 *ij*	0.691±0.059 *jk*	2.151±0.005 *j*	0.168-±0.081 *k*	- *i*	1.268±0.02 *fg*	1.366±0.115 *mn*	18.474±0.613 *g*	73.877±1.556 *ml*
Patriot	9.975±0.539 *m*	20.058±1.017 *b*	2.999±0.122 *hi*	10.492±0.471 *n*	5.903±0.316 *a*	6.951±0.316 *no*	3.242±0.332 *c*	15.498±0.196 *b*	1.846±0.812 *kl*	4.368±0.009 *a*	15.628±0.202 *o*	1.644±0.074 *c*	29.202±1.431 *a*	8.936±0.546 *m*	136.742±6.378 *j*
Puru	12.975±0.159 *l*	6.146±0.106 *k*	2.335±0.038 *k*	17.717±0.051 *j*	1.14±0.010 *h*	8.802±0.012 *l*	2.173±0.192 *jk*	4.084±0.012 *i*	1.170±0.087 *n*	0.893±0.074 *fg*	14.775±0.053 *o*	1.007±0.165 *i-k*	6.008±0.005 *j*	10.663±0.164 *l*	89.888±1.122 *o*
Reka	18.127±0.606 *j*	9.366±0.251 *h*	3.002±0.105 *hi*	21.006±0.608 *hi*	1.700±0.007 *e*	11.647±0.007 *j*	2.761±0.361 *fg*	6.345±0.065 *g*	1.604±0.074 *lm*	1.348±0.049 *e*	21.305±0.066 *l-n*	1.055±0.028 *h-j*	10.046±0.208 *gh*	14.487±0.553 *ij*	123.798±2.982 *k*
Spartan	16.422±0.763 *k*	12.42±0.638 *f*	2.002±0.102 *lm*	16.809±0.807 *jk*	1.442±0.027 *f*	10.125±0.027 *k*	1.402±0.429 *no*	9.083±0.021 *e*	0.856±0.336 *op*	1.300±0.086 *e*	23.020±0.042 *l*	0.637±0.023 *n*	17.338±0.705 *e*	13.747±0.502 *j*	126.603±4.506 *k*
Sunrise	53.189±1.260 *a*	1.334±0.002 *m*	6.916±0.137 *a*	37.252±0.841 *a*	0.508±0.011 *jk*	34.514±0.011 *a*	4.536±1.038 *b*	1.121±0.092 *jk*	4.788±0.006 *a*	0.684±0.090 *f-i*	71.609±0.072 *a*	1.981±0.028 *ab*	2.234±0.019 *kl*	31.355±0.541 *a*	252.022±4.148 *a*
Sweetheart	10.657±0.335 *m*	7.610±0.188 *j*	3.287±0.069 *f*	11.435±0.380 *n*	2.490±0.092 *c*	7.755±0.092 *l-n*	3.184±0.235 *cd*	5.344±0.125 *h*	2.115±0.175 *j*	2.294±0.069 *d*	19.627±0.061 *mn*	1.459±0.085 *de*	10.112±0.236 *gh*	12.049±0.363 *k*	99.417±2.501 *mn*

Data were recorded as the mean ± SD (n=3)(mg/100 g). ANOVA and Duncan test was performed. When $p < 0.05$, the values of the same column which are significantly different are indicated by different letters

Table S5: Level comparison of antioxidant capacity among 26 blueberry cultivars.

Cultivars	FRAP	ABTS
	μmol $FeSO_4$/g	umol Trolox/g
Duke	65.7±6.2^{gh}	28.0±0.2^{a}
Amblue	67.5±2.2^{gh}	27.7±1.4^{a}
Bluechip	137.9±0.2^{a}	20.0±0.8^{bcde}
Bluecrop	83.1±3.7^{cde}	18.5±1.2^{cdefg}
Bluegold	120.1±9.8^{b}	24.1±3.3^{abc}
Bluehaven	38.1±5.5^{mno}	13.2±2.5^{hg}
Bluejay	45.1±7.7^{lmn}	25.4±2.4^{ab}
Bluetta	48.8±2.1^{kl}	21.6±2.6^{bcd}
Bonifacy	67.3±2.3^{gh}	15.3±2.1^{efgh}
Brigitta	45.8±7.5^{klm}	12.9±0.3^{h}
Coville	55.0±7.5^{ijkl}	20.9±1.3^{bcde}
Earliblue	93.0±5.5^{c}	25.3±0.3^{ab}
Elliott	35.7±6.7^{no}	28.7±4.7^{a}
Hardyblue	81.1±5.1^{def}	19.2±5.1^{cdef}
Jersey	54.3±5.5^{jkl}	20.6±1.1^{bcde}
Jubilee	64.6±4.0^{hi}	16.1±0.2^{defgh}
Lateblue	20.8±3.3^{p}	19.0±0.6^{cdef}
Misty	55.6±3.0^{ijk}	14.4±0.6^{fgh}
Olimpia	29.8±2.8^{op}	16.1±4.8^{defgh}
O'Neal	85.6±2.0^{cd}	13.1±2.0^{gh}
Patriot	68.4±0.4^{gh}	15.6±7.1^{efgh}
Puru	74.9±5.5^{efg}	16.3±2.8^{defgh}
Reka	66.6±1.9^{gh}	17.9±3.4^{defgh}
Spartan	68.8±1.4^{gh}	23.9±2.2^{bca}
Sunrise	62.2±2.4^{hij}	28.6±0.2^{a}
Sweetheart	71.8±5.5^{fgh}	18.0±1.6^{defgh}

Data were recorded as the mean ± SD (n=3). ANOVA and the Duncan test was performed. When $p < 0.05$, the values of the same column which are significantly different are indicated by different letters.
